# New York Heart Association Class and Kansas City Cardiomyopathy Questionnaire in Acute Heart Failure

**DOI:** 10.1001/jamanetworkopen.2023.39458

**Published:** 2023-10-24

**Authors:** Xiqian Huo, Boxuan Pu, Wei Wang, Yue Peng, Jingkuo Li, Lubi Lei, Lihua Zhang, Jing Li

**Affiliations:** 1 National Clinical Research Center for Cardiovascular Diseases, State Key Laboratory of Cardiovascular Disease, Fuwai Hospital, National Center for Cardiovascular Diseases, Chinese Academy of Medical Sciences and Peking Union Medical College, Beijing, People's Republic of China

## Abstract

**Importance:**

Sparse data exist regarding how clinician-assigned New York Heart Association (NYHA) class compares with heart failure (HF)–specific Kansas City Cardiomyopathy Questionnaire (KCCQ) in acute HF.

**Objective:**

To compare concordance between NYHA class and KCCQ overall summary score (KCCQ-OS) in acute HF and investigate associations of changes in NYHA class and KCCQ-OS with long-term outcomes.

**Design, Setting, and Participants:**

In this cohort study, patients with HF were enrolled from 52 hospitals in China between August 2016 and May 2018. Among patients with NYHA class and KCCQ-OS at admission and 1 month, levels of each scale were categorized into 4 groups from worst to best. Mild and moderate to severe discordance were defined as NYHA class and KCCQ-OS differing by 1 level or 2 or more levels, respectively. Multivariable models evaluated associations between improvements in the 2 measures and outcomes. Analysis was conducted from January to March 2023.

**Exposure:**

Changes in NYHA class and KCCQ-OS from admission to 1 month.

**Main Outcomes and Measures:**

All-cause mortality, cardiovascular death, or first HF rehospitalization.

**Results:**

A total of 2683 patients (1709 [63.7%] male; median [IQR] age, 66 [56-75] years) were included. NYHA class II, III, and IV were presented in 374 patients (13.9%), 1179 patients (44.0%), and 1130 patients (42.1%), respectively, and the median (IQR) KCCQ-OS was 44.4 (28.3-61.9). Concordance, mild discordance, and moderate to severe discordance between admission NYHA class and KCCQ-OS occurred in 954 patients (35.6%), 1203 patients (44.8%), and 526 patients (19.6%), respectively. For KCCQ-OS, kernel density overlaps were 73.6% between NYHA II and III, 63.8% between NYHA II and IV, and 88.3% between NYHA III and IV. Most patients experienced improvements in NYHA and KCCQ-OS from admission to 1 month. After adjustment, there was no significant association between improvements in NYHA class and 4-year all-cause mortality, whereas 5 or more point improvements in KCCQ-OS were independently associated with a lower risk of 4-year mortality (hazard ratio, 0.84; 95% CI, 0.74-0.96; *P* = .01). NYHA class and KCCQ-OS improvements were both associated with decreased risk of 1-year composite cardiovascular death or HF rehospitalization.

**Conclusions and Relevance:**

In this cohort study of acute HF, discordance between NYHA class and KCCQ was common, and KCCQ was more relevant to subsequent mortality than NYHA class.

## Key Points

**Question:**

How is clinician-assigned New York Heart Association (NYHA) classification concordant with patient-reported Kansas City Cardiomyopathy Questionnaire (KCCQ), and are changes in NYHA and KCCQ associated with outcomes in acute heart failure?

**Findings:**

In this cohort study of 2683 patients, nearly half exhibited mild discordance, and 1 in 5 exhibted moderate to severe discordance between NYHA class and KCCQ overall summary (KCCQ-OS) at admission. Changes in KCCQ-OS were not reliably reflected in NYHA, and an improvement in KCCQ-OS was independently associated with a lower risk of 4-year mortality, while improvement in NYHA class was not.

**Meaning:**

These findings suggest KCCQ may be more sensitive for assessing health status changes and estimating outcomes in heart failure and can be integrated with NYHA class in clinical practice.

## Teaser

This cohort study evaluates concordance between New York Heart Association class and Kansas City Cardiomyopathy Questionnaire overall summary score among patients with acute heart failure in China.

## Introduction

The New York Heart Association (NYHA) classification has been widely used for heart failure (HF) symptom severity assessment since the 1920s.^1^ It is ingrained in guidelines and clinical practice, and NYHA class has served as the benchmark for risk stratification, trial enrollment, and treatment candidacy determination.^2^ However, there has been recognition of its potential limitations (eg, marked interobserver variability and limited reproducibility),^3,4^ and its prognostic relevance has been questioned increasingly.^5,6^ Since the inception of NYHA class, patient-reported outcome (PRO) measures have evolved substantially. The Kansas City Cardiomyopathy Questionnaire (KCCQ) is an extensively validated PRO instrument for quantitatively measuring HF-specific health status.^7,8,9,10,11^

However, comparisons between the KCCQ and NYHA classification among patients with acute HF in clinical practice remain unknown. Few studies have reported the prognostic value of PROs compared with NYHA and their conflicts, and the results have been affected by some limitations, such as inclusion of patients exclusively with chronic HF with reduced ejection fraction,^6^ short-term outcomes, and use of a general health survey rather than an HF-specific instrument (eg, KCCQ).^12^ In 2015, a shorter version of KCCQ, KCCQ-12, reduced the original 23-item questionnaire to 12 items, taking half the time to complete and increasing feasibility, particularly in acute care settings. Given the rapid progress of symptom severity in HF, it would be informative to compare the concordance in NYHA class and KCCQ-12 and the long-term outcome values associated with their early changes. Accordingly, using data from a nationwide cohort of patients with acute HF, we aimed to (1) characterize the level of concordance between admission NYHA class and KCCQ and assess correlations between them, (2) describe patterns of changes in NYHA class and KCCQ over time, and (3) compare the association of changes in NYHA class and KCCQ for long-term outcomes.

## Methods

### Study Design and Population

We included patients who were enrolled in the China Patient-Centered Evaluative Assessment of Cardiac Events prospective HF study and had NYHA class and KCCQ data at admission and 1-month follow-up. The study enrolled patients hospitalized for HF between August 2016 and May 2018 from 52 diverse hospitals located in 20 provinces.^13^ Patients hospitalized for HF in these hospitals were consecutively registered if they were aged 18 years or older. Those who signed the informed consent were enrolled and followed up at 1, 6, and 12 months after discharge and annually thereafter until March 2022. The ethics committee of Fuwai Hospital and the local ethics committees at sites approved the study. The study followed the Strengthening the Reporting of Observational Studies in Epidemiology (STROBE) reporting guidelines.^14^

### Data Collection

Patients’ demographics, socioeconomic status, depressive symptoms, and cognitive function were collected by standardized questionnaire through interviews in person during hospitalization. Clinical characteristics (eg, systolic blood pressure [SBP] and heart rate), comorbidities, and treatments were obtained from the medical records of the index hospitalization. Left ventricular ejection fraction (LVEF) was uniformly measured during hospitalization, and patients were categorized into HF with reduced ejection fraction (LVEF ≤40%), HF with mildly reduced ejection fraction (LVEF 41%-49%), and HF with preserved ejection fraction (LVEF ≥50%). Laboratory tests at admission, including N-terminal pro-B type natriuretic peptide (NT-proBNP), creatinine, sodium, and potassium were analyzed at the central laboratory. The estimated glomerular filtration rate (eGFR) was calculated according to the adaptation of the Modification of Diet in Renal Disease.^15^ Depression was evaluated by the Patient Health Questionnaire-2,^16^ and cognitive function was assessed by the Mini-Cog test.^17^

### NYHA Class and HF-Specific KCCQ Assessment

NYHA class was a prespecified 4-tier schema of class I to IV and assessed by clinicians at admission and 1 month. NYHA class IV indicates the worst functional status in HF and vice versa.^7^ The HF-specific PRO was measured within 48 hours of index admission and 1 month by KCCQ-12, which quantifies 4 health status domains: symptom frequency, physical limitations, social limitations, and quality of life.^11^ The KCCQ overall summary score (KCCQ-OS) was generated from the previously listed domains, ranging from 0 (worst) to 100 (best).

### Definition of Concordance and Discordance

We defined concordance and discordance through an established framework using cut points to categorize NYHA class and KCCQ-OS. Each scale was categorized into 4 levels from worst to best, and cut points for NYHA class (class IV, III, II, I) and KCCQ-OS (<25, 25-49, 50-74, 75-100) were chosen to be consistent with previous literature.^6^ Concordance was defined as having NYHA class and KCCQ-OS at the same level among these categories. Mild and moderate to severe discordance were defined by the level of NYHA class and KCCQ-OS differing by 1 level or 2 or more levels, respectively.

### Outcomes

The main outcome was 4-year all-cause mortality. Other outcomes included 1-year all-cause mortality and the composite of cardiovascular (CV) death or first rehospitalization for HF. CV death was defined as sudden cardiac death, or death due to HF, cerebrovascular events, coronary heart disease, or other CV causes. Death events and underlying reasons were collected from death certificates, patient’s relative interviews, or the national database of death causes, then centrally adjudicated by trained clinicians.

### Statistical Analysis

Patients’ characteristics stratified by level of discordance between admission NYHA class and KCCQ-OS (concordance, mild discordance, and moderate to severe discordance) were compared using the Kruskal-Wallis tests for continuous variables and χ^2^ tests for categorical variables. The difference between groups was calculated by standardized mean difference (SMD), and absolute values less than 0.1 were considered small differences.

Kernel density estimations were used to describe the distribution of KCCQ-OS by NYHA class. Silverman rule-of-thumb was applied for bandwidth selection.^18^ The overlap between classes was defined as the area of the intersection divided by the total area under both curves to quantify the similarity between distributions.^19^ We used Spearman correlation to compute the correlation coefficients between NYHA class and KCCQ with its domains at admission and 1 month.

Changes in NYHA class and KCCQ-OS between admission and 1 month were summarized in categorical and continuous analyses. Considering that a change of 5 or more points in KCCQ-OS is clinically significant, changes in KCCQ-OS were categorized into 10 or more points decline, 5 to 9 points decline, no significant change (<5-point change), 5 to 9 points improvement, and 10 or more points improvement. A logistic regression model was used to determine patient factors associated with specific discordance directionality between NYHA class and KCCQ-OS at admission and 1 month. Model selection was based on backward elimination, and variables with a *P* value greater than .05 were removed.

Cox proportional hazard models were used to separately evaluate the associations of improvements in NYHA class and improvements of 5 or more points in KCCQ-OS with outcomes. We adjusted the following variables in the models: age, sex, educational attainment, employment, smoking, depression status, cognitive function, SBP, NT-proBNP, eGFR, serum sodium, potassium, LVEF subtypes, atrial fibrillation, diabetes, chronic obstructive pulmonary disease (COPD), myocardial infarction, stroke, anemia, prior HF, cardiac resynchronization therapy (CRT), implantable cardioverter-defibrillator (ICD), and postdischarge medications. A sensitivity analysis was performed to evaluate whether improvements of 10 and 20 points in KCCQ-OS, which were considered moderate and large clinical changes,^20^ were associated with outcomes. We conducted subgroup analyses to examine the consistency of the associations. The subgroup parameters included age, gender, HF subtype (ie, new-onset HF or acute decompensated chronic HF), LVEF, NT-proBNP, eGFR, atrial fibrillation, diabetes, and COPD.

In this study, missing variables ranged from 0% to 4.73% (ie, eGFR, NT-proBNP, LVEF, sodium, and potassium) as presented in eTable 1 in Supplement 1 and were imputed using multiple imputations by the Markov chain Monte Carlo method. All comparisons were 2-sided, and statistical significance was defined as *P* < .05. We performed statistical analyses using SAS version 9.4 (SAS Institute). Analysis was conducted from January to March 2023.

## Results

### Patient Characteristics

In total, 2683 patients were included in this analysis (eFigure 1 and eTable 2 in Supplement 1). Their median (IQR) age was 66 (56-75) years, and 1709 patients (63.7%) were male. At admission, 374 (13.9%) were NYHA class II, 1179 (44.0%) class III, and 1130 (42.1%) class IV. The median (IQR) KCCQ-OS was 44.4 (28.3-61.9), 314 patients (11.7%) scored 75 to 100, 804 (30.0%) scored 50 to 74, 1021 (38.0%) scored 25 to 49, and 544 (20.3%) scored less than 25 (eTable 3 and eTable 4 in Supplement 1).

### Discordance Between NYHA Class and KCCQ-OS

At admission, 954 patients (35.6%) had concordant NYHA class and KCCQ-OS, 1203 (44.8%) had mild discordance, and 526 (19.6%) had moderate to severe discordance. Compared with patients with concordance, those with mild or moderate to severe discordance were younger; less frequently female; more likely to have higher educational attainment and employment, HF with reduced ejection fraction, depression, and stroke; more likely to have worse NYHA classification; and more likely to have a better KCCQ-OS score and EuroQoL 5-dimension index (EQ-5D) (Table 1).

**Table 1.** Admission Characteristics by the Degree of Admission Discordance Between NYHA and KCCQ-OS

**Characteristic**	Patients, No. (%)				
	Total (N = 2683)	Concordance (n = 954)	Mild discordance (n = 1203)	Moderate to severe discordance (n = 526)	SMD^a^
Age, median (IQR), y	66 (56-75)	67 (57-76)	66 (56-74)	63 (53-72)	0.185
Sex					
Male	1709 (63.7)	572 (60.0)	760 (63.2)	377 (71.7)	0.166
Female	974 (36.3)	382 (40.0)	443 (36.8)	149 (28.3)	
Educational status					
Primary school or below	1024 (38.2)	389 (40.8)	462 (38.4)	173 (32.9)	0.124
Middle school	828 (30.8)	293 (30.7)	354 (29.4)	181 (34.4)	
High school or above	831 (31.0)	272 (28.5)	387 (32.2)	172 (32.7)	
Employed	501 (18.7)	141 (14.8)	225 (18.7)	135 (25.7)	0.182
Married	2181 (81.3)	763 (80.0)	973 (80.9)	445 (84.6)	0.081
Clinical characteristics					
SBP, median (IQR), mm Hg	130 (118-149)	130 (117-147)	130 (118-148)	135 (118-150)	0.098
DBP, median (IQR), mm Hg	80 (70-90)	80 (70-90)	80 (70-90)	80 (71-95)	0.147
Heart rate, median (IQR), bpm	86 (73-100)	87 (73-102)	87 (73-100)	85 (73-100)	0.010
LVEF, %, median (IQR)	43 (33-55)	44 (34-57)	44 (33-56)	41 (33-50)	0.165
LVEF subtypes, No. (%)					
HFrEF	1254 (46.7)	438 (45.9)	546 (45.4)	270 (51.3)	0.173
HFmrEF	533 (19.9)	167 (17.5)	244 (20.3)	122 (23.2)	
HFpEF	896 (33.4)	349 (36.6)	413 (34.3)	134 (25.5)	
Current smoker, No. (%)	737 (27.5)	240 (25.3)	320 (26.6)	176 (33.5)	0.120
Depression status, No. (%)	1606 (59.9)	653 (68.5)	725 (60.3)	228 (43.4)	0.346
Cognitive impairment, No. (%)	791 (29.5)	312 (32.7)	340 (28.3)	139 (26.4)	0.092
NYHA class, No. (%)					
II	374 (13.9)	152 (16.0)	194 (16.1)	28 (5.3)	0.529
III	1179 (44.0)	487 (51.0)	560 (46.6)	132 (25.1)	
IV	1130 (42.1)	315 (33.0)	449 (37.3)	366 (69.6)	
Medical history					
Hypertension	1590 (59.3)	548 (57.4)	723 (60.1)	319 (60.6)	0.043
Atrial fibrillation	957 (35.7)	367 (38.5)	424 (35.2)	166 (31.6)	0.097
Myocardial infarction	636 (23.7)	230 (24.1)	277 (23.0)	129 (24.5)	0.023
Diabetes	854 (31.8)	299 (31.3)	384 (31.9)	171 (32.5)	0.017
COPD	495 (18.4)	181 (19.0)	235 (19.5)	79 (15.0)	0.080
Stroke	542 (20.2)	220 (23.1)	238 (19.8)	84 (16.0)	0.120
Anemia	447 (16.7)	178 (18.7)	199 (16.5)	70 (13.3)	0.098
New-onset HF	767 (28.6)	246 (25.8)	347 (28.8)	174 (33.1)	0.107
Biomarkers at admission, median (IQR)					
NT-proBNP, ng/L	1323 (555-2870)	1429 (593-3119)	1268 (507-2875)	1266 (516-2664)	0.067
Sodium, mmol/L	140 (138-142)	140 (137-142)	140 (138-142)	140 (137-142)	0.058
Potassium, mmol/L	4.1 (3.7-4.4)	4.1 (3.8-4.4)	4.1 (3.7-4.4)	4.0 (3.7-4.3)	0.078
eGFR, mL/min/1.73m^2^	74 (59-89)	73 (58-88)	74 (59-90)	75 (59-91)	0.064
CRT/ICD	15 (0.6)	6 (0.6)	8 (0.7)	1 (0.2)	0.049
Treatment at discharge					
ACEI or ARB	1421 (53.0)	493 (51.7)	652 (54.2)	276 (52.5)	0.034
β-Blockers	1646 (61.3)	586 (61.4)	726 (60.3)	334 (63.5)	0.043
Aldosterone antagonists	1731 (64.5)	630 (66.0)	766 (63.7)	335 (63.7)	0.033
Quality of life, median (IQR)					
KCCQ-OS	44.4 (28.3-61.9)	34.0 (19.8-45.8)	43.1 (29.8-60.4)	67.1 (56.7-80.2)	1.200
EQ-5D index	0.7 (0.5-0.8)	0.6 (0.3-0.8)	0.7 (0.5-0.8)	0.8 (0.7-0.9)	0.541
EQ-5D VAS	60 (50-75)	60 (50-70)	60 (50-70)	70 (60-80)	0.392

Abbreviations: ACEI, angiotensin-converting enzyme inhibitor; ARB, angiotensin receptor blocker; bpm, beats per minute; COPD, chronic obstructive pulmonary disease; CRT, cardiac resynchronization therapy; DBP, diastolic blood pressure; eGFR, estimated glomerular filtration rate; EQ-5D, EuroQoL 5-dimension; HF, heart failure; HFmrEF, heart failure with mildly reduced ejection fraction; HFpEF, heart failure with preserved ejection fraction; HFrEF, heart failure with reduced ejection fraction; ICD, implantable cardioverter defibrillator; KCCQ-OS, Kansas City Cardiomyopathy Questionnaire overall summary score; LVEF, left ventricular ejection fraction; NT-proBNP, N-terminal pro-B type natriuretic peptide; NYHA, New York Heart Association; SBP, systolic blood pressure; SMD, standardized mean difference; VAS, visual analog scale.

To convert potassium to milliequivalents per liter, multiply by 1.0; to convert sodium to milliequivalents per liter, multiply by 1.0

^a^ SMDs less than 0.10 are considered small.

Proportions of patients with varying severity of discordance between NYHA class and KCCQ-OS are depicted in eTable 5 in Supplement 1. At admission, the following characteristics were each associated with a lower likelihood of the KCCQ-OS being better than the NYHA class: female, employed, depression, COPD, stroke, and HF with preserved ejection fraction. At 1 month, being female, working, and having depression were still associated with a lower likelihood of KCCQ-OS being better than NYHA class, with the addition of being married and having anemia. Factors associated with the likelihood of NYHA class outperforming KCCQ-OS are also summarized in eTable 6 in Supplement 1.

### Correlations Between NYHA Class and KCCQ Measurements

**Figure 1.**
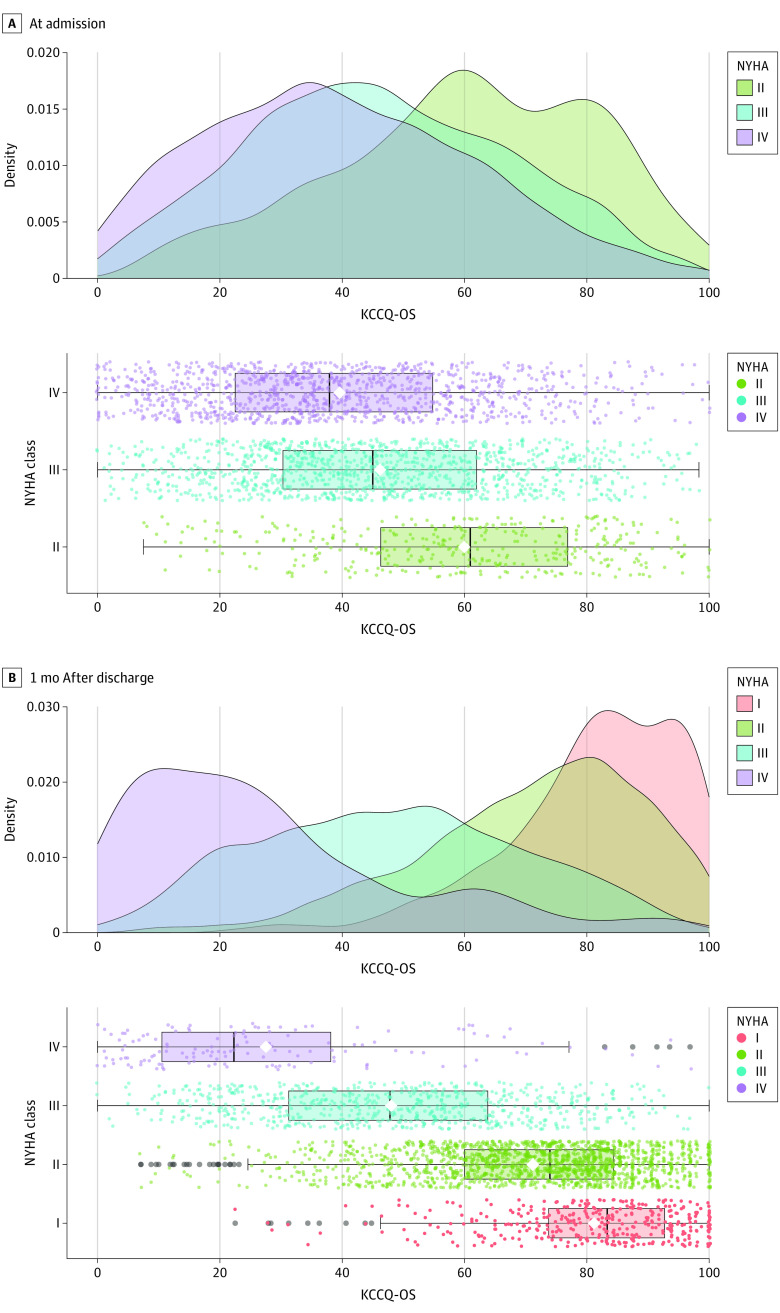
Distribution of KCCQ-OS by NYHA Class at Admission and 1 Month Data points represent individual patients, with the corresponding density plots displayed above for admission Kansas City Cardiomyopathy Questionnaire overall summary (KCCQ-OS) score (panel A) and 1-month KCCQ-OS score (panel B). Box plots display median (central vertical line), mean (central white diamond), IQR (vertical lines on box edges), and value no further than 1.5 × IQR from the edges of the box (whiskers and outer vertical lines). NYHA indicates New York Heart Association.

The distributions of KCCQ-OS levels stratified by admission and 1-month NYHA class are presented in Figure 1. At admission, for KCCQ-OS, kernel density overlaps were 73.6% between NYHA II and III, 63.8% between NYHA II and IV, and 88.3% between NYHA III and IV. At 1 month, overlaps between NYHA class for KCCQ-OS varied from 20.9% (NYHA I vs IV) to 76.2% (NYHA I vs II). The correlations between NYHA class and KCCQ-OS with its domains were statistically significant at admission and 1 month. The magnitude of the correlation between NYHA class and KCCQ-OS was modest (*r* = 0.26) at admission and higher (*r* = 0.54) at 1 month (all *P* < .001) (eTable 7 in Supplement 1).

### Change in NYHA Class and KCCQ From Admission to 1 Month

**Figure 2.**
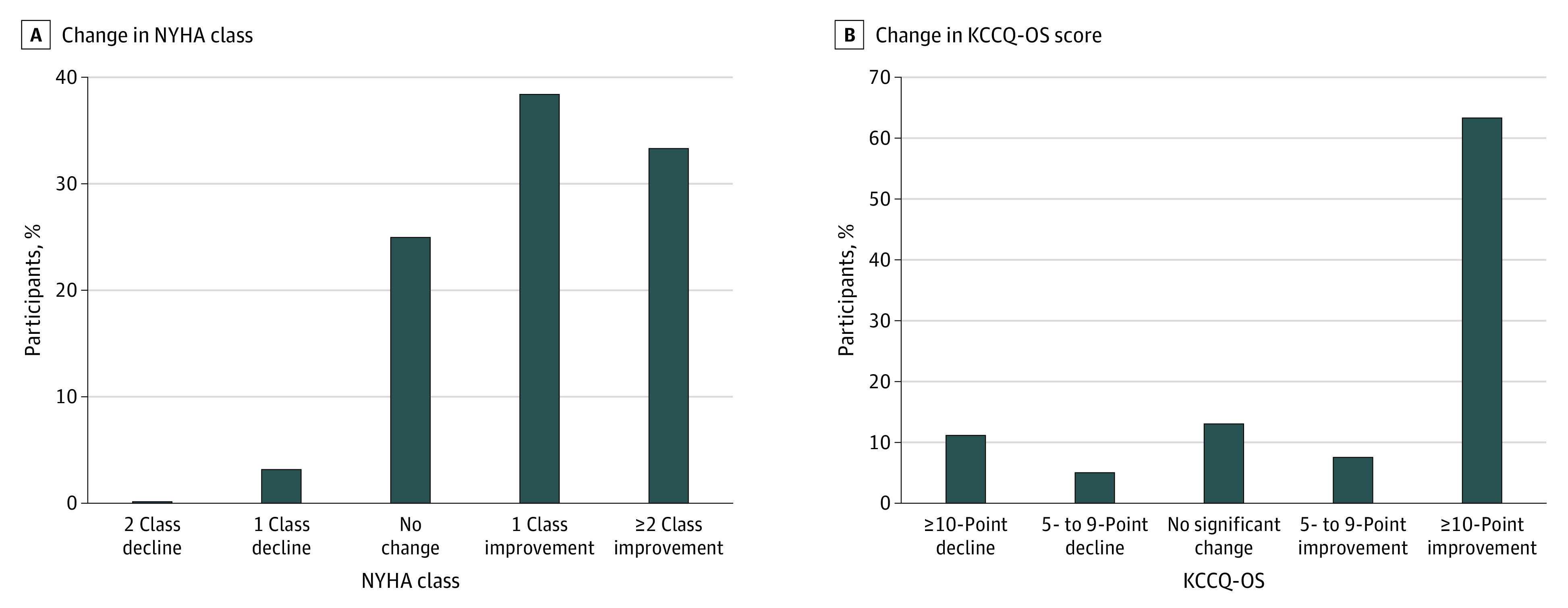
Change From Admission to 1 Month in NYHA Class and KCCQ-OS KCCQ-OS indicates Kansas City Cardiomyopathy Questionnaire overall summary; NYHA, New York Heart Association.

From admission to 1 month after discharge, 1924 patients (71.7%) experienced any improvement in NYHA class, 670 (25.0%) had no change, and 89 (3.3%) had any decline. For KCCQ-OS, the most common change was a 10-point or more improvement in 1699 patients (63.3%). A total of 204 patients (7.6%) had improvements of 5 to 9 points, 349 patients (13.0%) had no significant change, and 431 patients (16.1%) experienced a decline of 5 or more points (Figure 2).

Among patients with no change in NYHA class, the median (IQR) change in KCCQ-OS was 7 (−6 to 24). For patients with improvements of 2 or more NYHA classes, the median (IQR) change of KCCQ-OS was 29 (13 to 47). For those with a decline of 2 NYHA classes, KCCQ-OS reflected a median (IQR) change of −6 (−17 to 12), with KCCQ physical limitation score having the largest decline of −21 (−29 to 4) (Table 2).

**Table 2.** Change in KCCQ Score From Admission to 1 Month by the Change in NYHA Class

Change	Decline of 2 classes (n = 4)	Decline of 1 class (n = 85)	No change (n = 670)	Improvement of 1 class (n = 1030)	Improvement of ≥2 classes (n = 894)
KCCQ, median (IQR)					
OS	−6 (−17 to 12)	2 (−14 to 23)	7 (−6 to 24)	19 (3 to 34)	29 (13 to 47)
PLS	−21 (−29 to 4)	0 (−17 to 17)	0 (−8 to 25)	17 (0 to 33)	25 (6 to 42)
SFS	5 (−8 to 8)	5 (−10 to 25)	10 (−5 to 35)	25 (5 to 45)	35 (15 to 60)
QOLS	0 (−13 to 25)	0 (−13 to 1)	0 (−13 to 25)	13 (0 to 38)	25 (13 to 50)
SLS	0 (−21 to 25)	0 (−25 to 25)	0 (−8 to 25)	17 (0 to 42)	25 (0 to 50)

Abbreviations: KCCQ, Kansas City Cardiomyopathy Questionnaire; NYHA, New York Heart Association; OS, overall summary score; PLS, physical limitation score; QOLS, quality of life score; SFS, symptom frequency score; SLS, social limitation score.

### Associations of Change in NYHA Class and KCCQ With Outcomes

There were 1057 (39.4%) deaths during the 4-year follow-up, of which 275 patients (10.2%) died within 1 year after discharge, and 958 (35.7%) had 1-year CV death or HF rehospitalization. In unadjusted analysis, any improvement in NYHA class and a 5-point or more improvement in KCCQ-OS were both associated with lower risks of all-cause mortality and the composite of CV death or HF rehospitalization.

**Figure 3.**
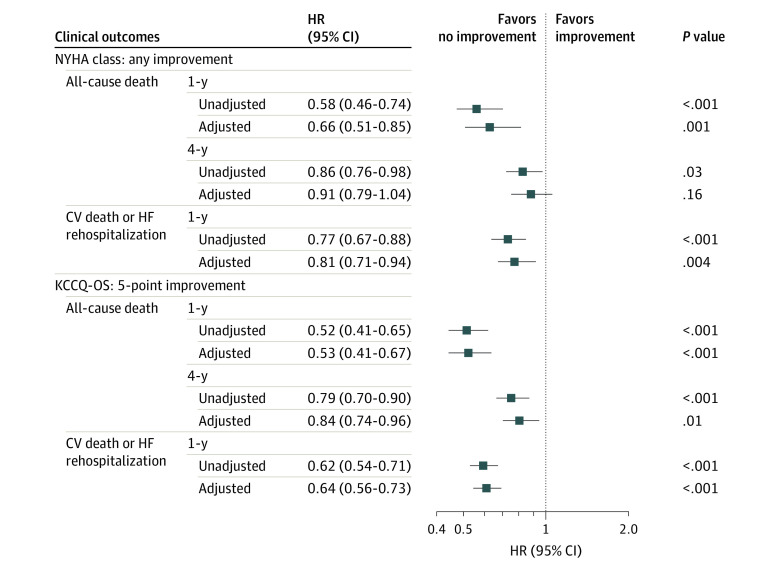
Association Between Change in NYHA Class and KCCQ-OS Score With Clinical Outcomes Unadjusted and adjusted hazard ratios indicate the risk of clinical outcomes associated with improvement in NYHA class and KCCQ-OS score. Models were adjusted for age, sex, educational attainment, employment, smoking, depression status, cognitive function, systolic blood pressure, N-terminal pro-B type natriuretic peptide, estimated glomerular filtration rate, serum sodium, serum potassium, left ventricular ejection fraction subtypes, atrial fibrillation, diabetes, chronic obstructive pulmonary disease, myocardial infarction, stroke, anemia, prior heart failure, cardiac resynchronization therapy, implantable cardioverter-defibrillator, and postdischarge medications. CV indicates cardiovascular; HF, heart failure; KCCQ-OS, Kansas City Cardiomyopathy Questionnaire overall summary; NYHA, New York Heart Association.

After adjustment for covariates, there was no significant association between improvement in NYHA class and 4-year all-cause mortality (hazard ratio [HR], 0.91; 95% CI, 0.79-1.04), whereas an improvement of 5 or more points in KCCQ-OS was independently associated with a 16% lower risk of 4-year mortality (HR, 0.84; 95% CI, 0.74-0.96). The estimation of the composite outcome of CV death and HF hospitalization was greater for an improvement of 5 or more points in KCCQ-OS (HR, 0.64; 95% CI, 0.56-0.73) than for an improvement in NYHA class (HR, 0.81; 95% CI, 0.71-0.94). Improvement in NYHA class (HR, 0.66; 95% CI, 0.51-0.85) and KCCQ-OS (HR, 0.53; 95% CI, 0.41-0.67) were both significantly associated with decreased risk of 1-year mortality (Figure 3). There were significant associations between improvements in each domain of KCCQ and clinical outcomes (eTable 8 in Supplement 1).

The sensitivity analyses showed similar associations of improvement of 10 or more or 20 points in KCCQ-OS with outcomes (eFigure 2 in Supplement 1). No significant heterogeneity was observed in the associations of improvement in NYHA class and KCCQ-OS with 4-year mortality in most subgroups as shown in eFigure 3 in Supplement 1, including age, sex, HF subtype, LVEF group, eGFR, atrial fibrillation, diabetes, and COPD.

## Discussion

In this contemporary nationwide cohort study of patients with acute HF, we found that about two-thirds of patients exhibited discordance between the NYHA class and KCCQ scale upon admission, with the vast majority of discordances related to a disproportionally better KCCQ-OS. Most patients experienced improvements in NYHA class and KCCQ-OS at 1 month after discharge, with substantial variability of KCCQ across NYHA class. KCCQ-OS improvement was independently associated with lower risks of all-cause mortality and the composite CV death or HF rehospitalization, whereas the change in NYHA class was not associated with 4-year mortality risk.

To our knowledge, this is the first study to provide a comparison between NYHA class and KCCQ-12 in patients with acute HF. Moderate to severe discordance between the NYHA class and KCCQ-OS was common in one-fifth of patients, and KCCQ was more sensitive for detecting changes in health status and estimating outcomes. One prior study^6^ reported that KCCQ’s prognostic value conflicted with NYHA class, but the evidence of comparisons was generated from stable patients with chronic HF with reduced ejection fraction. Another study^12^ explored the discordant relationship in the ASCEND-HF trial but used a general health survey (EQ-5D), and it was limited to short-term outcomes (ie, 30-day and 180-day mortality) and nonsimultaneous assessments (eg, NYHA was documented before decompensation whereas EQ-5D was reported after admission). Greater attention should be paid to the fact that symptoms reported by clinicians and patients often differ, and it may affect clinical decisions for optimal therapeutic options.

The present study was further strengthened by evaluating the changes in NYHA class and KCCQ status in long-term outcomes using data from a clinical practice-based acute HF population in China. Health status measurements for prognostication purpose have been reported in US and European trials,^3,21,22^ yet data on comparing NYHA and KCCQ changes among patients with HF in a clinical population setting are not well characterized, especially in developing countries where health status might vary substantially by socioeconomic status and social class.^23,24^ Additionally, considering symptoms change rapidly in acute HF, it would be of great practical importance to understand the patterns of early change in functional class after events and the impact of health status fluctuation on subsequent risk of events. Our data showed that improvements in KCCQ-OS were independently associated with the sheer magnitude of the 47% decreased risk of 1-year mortality and 16% decreased risk of 4-year mortality, which was amplified by the lack of a significant association between NYHA change and 4-year survival. Yet, despite a decade of advocacy for these measurements, health systems have been hesitating to adopt KCCQ within HF standard care due to various barriers.^25,26^ A recent trial of outpatients with HF showed that sharing KCCQ results with physicians enhanced the accuracy of health status assessment and boosted patients’ perception that clinicians understood their symptoms better.^27^ Integrating the clinician-assigned NYHA class and patient-reported KCCQ in routine care can lay the foundation for more efficient patient-centered care and improve health care quality.

The mechanisms underlying the disassociations between NYHA class and KCCQ scale remain unclear. Despite significant correlations, meaningful changes in KCCQ-12 were not reliably reflected in NYHA class. Possible explanations include interphysician variability in assessing NYHA class and different emphasis in various measurements. Similar conflicts between clinician-reported outcomes and PROs have been reported in other disease settings.^6,28,29^ For instance, Arnold and colleagues^28^ found 42% of patients with coronary disease had more frequent angina than those recognized by physicians, and a marked variation of 0%-86% in underrecognition across physicians was observed. Moreover, patients may be more willing to provide details on their symptoms when using self-reported questionnaires to ensure adequate attention is given. In many cases, clinicians tend to consider that patients are in less pain than they reported after taking patients’ medical history.^30,31^ Additionally, our study identified several patient factors, including sex, employment, marriage, psychological factors, and comorbidities such as COPD that were associated with disagreement between clinician-reported and patient-reported outcomes. Prior studies demonstrated that women reported poorer health status than men, and it is plausible that the gender gap may be attributed to factors such as biological, sociobehavioral, and psychological inequalities, and women may have more chronic conditions that are nonfatal but are debilitating to be linked with self-reported health problems.^32,33^ Likewise, current findings of employment and marriage status associated with discordance may suggest societal factors contributing to the clinician vs patient interpretation of symptoms.^23,34^ Furthermore, clinicians may tend to exclude breath shortness due to COPD from HF while patients do not. However, the role of other factors in driving disparities in care requires further investigation.

### Limitations

The study has some limitations. First, there is no exact mapping to equate what KCCQ score correlates to which NYHA class. However, the decision to use 4 equally sized 25-point ranges of KCCQ-OS was consistent with previous literature.^35^ Second, the assessment of NYHA class is subjective to floor and ceiling effects where patients with class I cannot improve and those with class IV cannot decline. Third, the associations of longitudinal changes in KCCQ and NYHA class over multiple time points for 4 years and clinical outcomes were not assessed as such data are not available in our study. Further studies are needed to examine the prognostic value of longitudinal changes in the measurements over a long period. Fourth, the study only included Chinese patients with complete data for NYHA class and KCCQ at admission and 1 month. Although generally similar characteristics were observed between the included and excluded patients, the proportion of unfavorable health status and discordance between the 2 measures may be underestimated and generalizability to other ethnicities needs further investigation.

## Conclusions

In this cohort study of acute HF, discordance between NYHA class and KCCQ was common. Compared with NYHA class, KCCQ-OS was more likely to be significantly associated with subsequent mortality, particularly with 4-year all-cause mortality. Efforts to integrate the patient’s voice in HF clinical practice and population health in combination with clinician-reported indices should be justified to improve health care quality and long-term outcomes.

## Notes

Supplement 1. **eTable 1. **Proportions of Missing Data in Covariates **eFigure 1. **Flow Chart of Study Cohort Development **eTable 2. **Characteristics of Patients Included and Excluded From Current Study **eTable 3. **Patient Characteristics by NYHA Class at Admission **eTable 4. **Patient Characteristics by KCCQ-OS at Admissio**n** **eTable 5. **Agreement Between NYHA Class and KCCQ-OS at Admission and 1 Month **eTable 6. **Factors Associated With Specific Directionality of Discordance Between NYHA Class and KCCQ-OS Categories **eTable 7. **Correlations Among NYHA Class and KCCQ Measurements **eTable 8. **Association Between Changes in Each Domain of KCCQ-OS and Clinical Outcomes **eFigure 2. **Sensitivity Analysis of the Association Between Change in KCCQ-OS and Clinical Outcomes **eFigure 3. **Subgroup Analysis of the Association Between Change in NYHA Class and KCCQ-OS With 4-Year All-Cause Mortality

Supplement 2.
Data Sharing Statement
