# Supplementary material for: New York Heart Association Class and Kansas City Cardiomyopathy Questionnaire in Acute Heart Failure
Source: JAMA Netw Open. 2023 Oct 24;6(10):e2339458. doi: 10.1001/jamanetworkopen.2023.39458 (PMC10599126; doi:10.1001/jamanetworkopen.2023.39458)
Supplement: Supplement 1. — eTable 1. Proportions of Missing Data in Covariates eFigure 1. Flow Chart of Study Cohort Development eTable 2. Characteristics of Patients Included and Excluded From Current Study eTable 3. Patient Characteristics by NYHA Class at Admission eTable 4. Patient Characteristics by KCCQ-OS at Admission eTable 5. Agreement Between NYHA Class and KCCQ-OS at Admission and 1 Month eTable 6. Factors Associated With Specific Directionality of Discordance Between NYHA Class and KCCQ-OS Categories eTable 7. Correlations Among NYHA Class and KCCQ Measurements eTable 8. Association Between Changes in Each Domain of KCCQ-OS and Clinical Outcomes eFigure 2. Sensitivity Analysis of the Association Between Change in KCCQ-OS and Clinical Outcomes eFigure 3. Subgroup Analysis of the Association Between Change in NYHA Class and KCCQ-OS With 4-Year All-Cause Mortality [file jamanetwopen-e2339458-s001.pdf]

Supplemental Online Content

Huo X, Pu B, Wang W, et a. New York Heart Association class and Kansas City Cardiomyopathy Questionnaire and acute heart failure. *JAMA Netw Open*. 2023;6(10):e2339458. doi:10.1001/jamanetworkopen.2023.39458

eTable 1. Proportions of Missing Data in Covariates
eFigure1. Flow Chart of Study Cohort Development
eTable 2. Characteristics of Patients Included and Excluded From Current Study
eTable 3. Patient Characteristics by NYHA Class at Admission
eTable 4. Patient Characteristics by KCCQ-OS at Admissio**n**
eTable 5. Agreement Between NYHA Class and KCCQ-OS at Admission and 1 Month
eTable 6. Factors Associated With Specific Directionality of Discordance Between NYHA Class and KCCQ-OS Categories
eTable 7. Correlations Among NYHA Class and KCCQ Measurements
eTable 8. Association Between Changes in Each Domain of KCCQ-OS and Clinical Outcomes
eFigure 2. Sensitivity Analysis of the Association Between Change in KCCQ-OS and Clinical Outcomes
eFigure 3. Subgroup Analysis of the Association Between Change in NYHA Class and KCCQ-OS With 4-Year All-Cause Mortality

This supplemental material has been provided by the authors to give readers additional information about their work.

© 2023 Huo X et al. *JAMA Network Open.*

eTable 1. Proportions of Missing Data in Covariates

Covariates	N	Missing (n, %)
NT-proBNP	2665	18 (0.7)
Sodium	2645	38 (1.4)
Potassium	2645	38 (1.4)
eGFR	2681	2 (0.1)
LVEF	2556	127 (4.7)

Abbreviation: NT-proBNP: N-terminal pro-B type natriuretic peptide; eGFR: estimated glomerular filtration rate; LVEF: left ventricular ejection fraction.

© 2023 Huo X et al. *JAMA Network Open.*

eFigure1. Flow Chart of Study Cohort Development

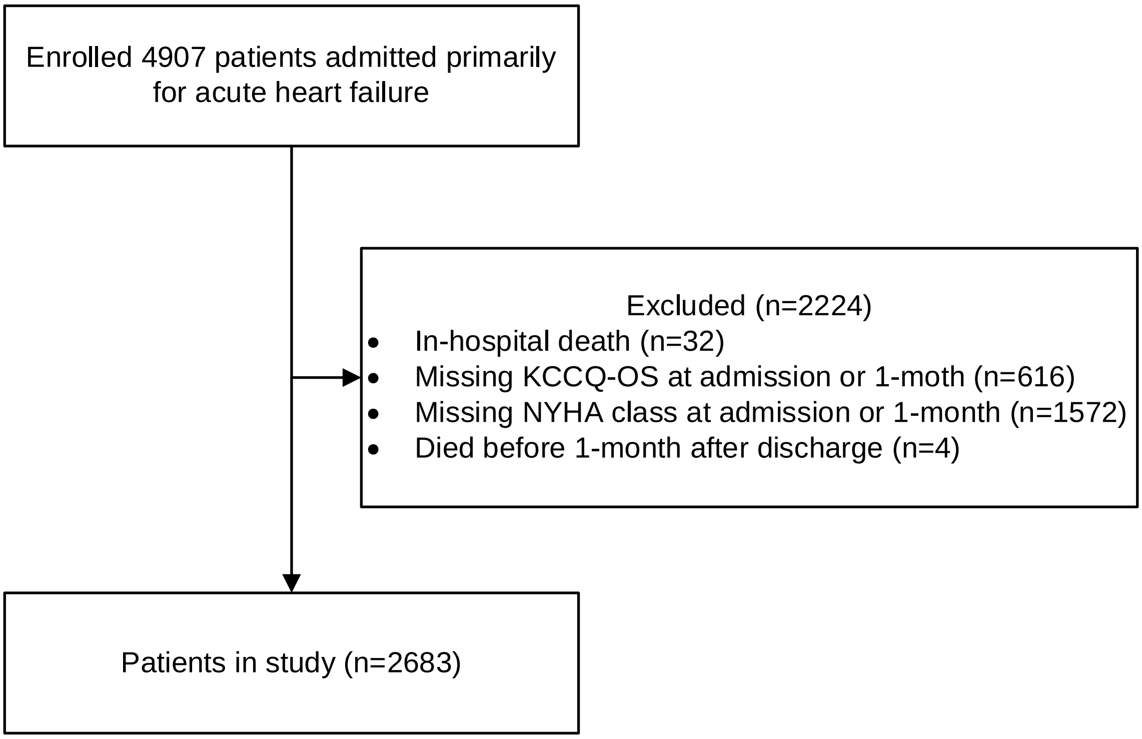

Abbreviation: KCCQ-OS: Kansas City Cardiomyopathy Questionnaire overall summary score; NYHA: New York Heart Association.

© 2023 Huo X et al. *JAMA Network Open.*

eTable 2. Characteristics of Patients Included and Excluded From Current Study

	Excluded (n=2224)	Included (n=2683)	SMD*
**Demographic**			
Age, years, median (IQR)	67(58-75)	66(56-75)	0.096
Sex, n (%)			0.015
Male	1433 (65.4)	1709 (63.7)	
Female	791 (35.6)	974 (36.3)	
Educational status, n (%)			0.194
Primary school or below	999 (47.6)	1024 (38.2)	
Middle school	530 (25.3)	828 (30.8)	
High school or above	569 (27.1)	831 (31.0)	
Employee, n (%)	1878 (84.4)	2182 (81.3)	0.083
Married, n (%)	493 (22.2)	502 (18.7)	0.086
**Clinical characteristics**			
SBP, mmHg, median (IQR)	130 (114-147)	130 (118-149)	0.094
DBP, mmHg, median (IQR)	80 (70-90)	80 (70-90)	0.078
Heart rate, bpm, median (IQR)	88 (76-100)	86 (73-100)	0.032
LVEF, %, median (IQR)	43 (32-56)	43 (33-55)	0.011
NT-proBNP, ng/L	1651 (736-3559)	1323 (555-2870)	0.113
**Medical history, n (%)**			
Hypertension	1264 (56.8)	1590 (59.3)	0.049
Atrial fibrillation	831 (37.4)	957 (35.7)	0.035
Myocardial infarction	424 (19.1)	636 (23.7)	0.113
Diabetes mellitus	627 (28.2)	854 (31.8)	0.079
COPD	372 (16.7)	495 (18.4)	0.045
Stroke	434 (19.5)	542 (20.2)	0.017
Depression	1394 (62.7)	1606 (59.9)	0.058
Cognitive impairment	880 (39.6)	791 (29.5)	0.213
**Medication at discharge, n (%)**			
ACEI/ARB	1272 (57.2)	1421 (53.0)	0.085
β-blockers	1422 (63.9)	1646 (61.3)	0.054
Aldosterone antagonists	1537 (69.1)	1731(64.5)	0.098
**Health status and quality of life**			

© 2023 Huo X et al. *JAMA Network Open.*

NYHA class, n (%)			0.111
II	300 (13.5)	374 (13.9)	
III	1093 (49.3)	1179 (43.9)	
IV	826 (37.2)	1130 (42.1)	
KCCQ-OS, median (IQR)	43.1 (27.7-58.8)	44.4 (28.3-61.9)	0.071
EQ-5D index, median (IQR)	0.7 (0.6-0.8)	0.7 (0.5-0.8)	0.008
EQ-5D VAS, median (IQR)	60 (50-75)	60 (50-75)	0.013
**Outcomes**			
LOS, day, median (IQR)	9 (7-12)	10 (7-3)	0.074
4-year all-cause mortaity	1062 (47.8)	1057 (39.4)	0.169

*SMDs less than 0.10 are considered small.

Abbreviation: SMD: standardized mean difference; IQR: interquartile range; SBP: systolic blood pressure; DBP: diastolic blood pressure; NYHA: New York Heart Association; COPD: chronic obstructive pulmonary disease; LVEF: left ventricular ejection fraction; NT-proBNP: N-terminal pro-B type natriuretic peptide; eGFR: estimated glomerular filtration rate; ACEI: angiotensin-converting enzyme inhibitor; ARB: angiotensin receptor blocker; KCCQ-OS: Kansas city Cardiomyopathy Questionnaire overall summary score; EQ-5D: EuroQoL 5-dimension; VAS: visual analog scale, LOS: length of stay.

.

© 2023 Huo X et al. *JAMA Network Open.*

eTable 3. Patient Characteristics by NYHA Class at Admission

	NYHA Class II (n=374)	NYHA Class III (n=1179)	NYHA Class IV (n=1130)	SMD*
**Demographic**				
Age, years, median (IQR)	66 (57-74)	66 (57-75)	65 (55-74)	0.050
Sex, n (%)				0.015
Male	238 (63.6)	745 (63.2)	726 (64.2)	
Female	136 (36.4)	434 (36.8)	404 (35.8)	
Educational status, n (%)				0.189
Primary school or below	112 (30.0)	451 (38.2)	461 (40.8)	
Middle school	124 (33.1)	339 (28.8)	365 (32.3)	
High school or above	138 (36.9)	389 (33.0)	304 (26.9)	
Employee, n (%)	66 (17.6)	220 (18.7)	215 (19.0)	0.024
Married, n (%)	310 (82.9)	976 (82.8)	895 (79.2)	0.063
**Clinical characteristics**				
SBP, mmHg, median (IQR)	130 (120-146)	130 (118-146)	132 (116-150)	0.080
DBP, mmHg, median (IQR)	78 (70-87)	80 (70-90)	80 (70-95)	0.214
Heart rate, bpm, median (IQR)	79 (66-92)	85 (72-100)	90 (78-105)	0.344
**Medical history, n (%)**				
Hypertension	230 (61.5)	699 (59.3)	661 (58.5)	0.041
Atrial fibrillation	111 (29.9)	430 (36.5)	415 (36.7)	0.096

© 2023 Huo X et al. *JAMA Network Open.*

Myocardial infarction	105 (28.1)	252 (21.4)	279 (24.7)	0.104
Diabetes mellitus	100 (26.7)	379 (32.1)	375 (33.2)	0.094
COPD	74 (19.8)	241 (20.4)	180 (15.9)	0.078
Stroke	85 (22.7)	253 (21.5)	204 (18.1)	0.077
Anemia	51 (13.6)	184 (15.6)	212 (18.8)	0.093
New-onset HF	153 (40.9)	338 (28.7)	276 (24.4)	0.237
Current smoking	91 (24.3)	298 (25.3)	348 (30.8)	0.097
Depression	215 (57.5)	692 (58.7)	699 (61.9)	0.059
Cognitive impairment	76 (20.3)	338 (28,7)	377 (33.4)	0.198
**LVEF, %, median (IQR)**	52 (40-62)	44 (33-56)	40 (31-50)	0.443
**LVEF subtypes, n (%)**				0.399
HFrEF	120 (32.1)	516 (43.7)	618 (54.7)	
HFmrEF	60 (16.0)	234 (19.9)	239 (21.1)	
HFpEF	194 (51.9)	429 (36.4)	273 (24.2)	
**Biomarkers at admission, median (IQR)**				
NT-proBNP, ng/L	588 (201-1352)	1207 (488-2641)	1863 (839-3725)	0.287
Sodium, mmol/L	140 (138-142)	140 (138-142)	140 (137-142)	0.089
Potassium, mmol/L	4.0 (3.7-4.4)	4.1 (3.8-4.4)	4.1 (3.7-4.4)	0.020
eGFR, mL/min/1.73m^2	77 (63-92)	74 (59-89)	73 (57-88)	0.129
**Medication at discharge, n (%)**				
ACEI/ARB	196 (52.4)	640 (54.3)	585 (51.8)	0.034

© 2023 Huo X et al. *JAMA Network Open.*

β-blockers	241 (64.4)	747 (63.4)	658 (58.2)	0.085
Aldosterone antagonists	204 (54.5)	771 (65.4)	756 (66.9)	0.170
**Quality of life at admission, median (IQR)**				
KCCQ-OS	60.9 (46.3-76.9)	45.0 (30.2-62.1)	37.9 (22.5-54.8)	0.629
EQ-5D index	0.8 (0.7-0.8)	0.7 (0.6-0.8)	0.6 (0.3-0.8)	0.404
EQ-5D VAS	70 (60-80)	60 (50-70)	60 (50-70)	0.294

*SMDs less than 0.10 are considered small.

Abbreviation: HF: heart failure; IQR: interquartile range; SMD: standardized mean difference; SBP: systolic blood pressure; DBP: diastolic blood pressure; NYHA: New York Heart Association; COPD: chronic obstructive pulmonary disease; LVEF: left ventricular ejection fraction; HFrEF: heart failure with reduced ejection fraction; HFmrEF: heart failure with mildly reduced ejection fraction; HFpEF: heart failure with preserved ejection fraction; NT-proBNP: N-terminal pro-B type natriuretic peptide; eGFR: estimated glomerular filtration rate; ACEI: angiotensin-converting enzyme inhibitor; ARB: angiotensin receptor blocker; KCCQ-OS: Kansas city Cardiomyopathy Questionnaire overall summary score; EQ-5D: EuroQoL 5-dimension; VAS: visual analog scale.

© 2023 Huo X et al. *JAMA Network Open.*

eTable 4. Patient Characteristics by KCCQ-OS at Admission

	KCCQ-OS 75-100 (n=314)	KCCQ-OS 50-74 (n=804)	KCCQ-OS 25-49 (n=1021)	KCCQ-OS 0-24 (n=544)	SMD*
**Demographic**					
Age, years, median (IQR)	63 (53-72)	64 (54-73)	67 (58-75)	67 (58-77)	0.192
Sex, n (%)					0.225
Male	230 (73.2)	558 (69.4)	623 (61.0)	298 (54.8)	
Female	84 (26.8)	246 (30.6)	398 (39.0)	246 (45.2)	
Educational status, n (%)					0.258
Primary school or below	96 (30.6)	257 (32.0)	409 (40.0)	262 (48.2)	
Middle school	88 (28.0)	277 (34.4)	309 (30.3)	154 (28.3)	
High school or above	130 (41.4)	270 (33.6)	303 (29.7)	128 (23.5)	
Employee, n (%)	80 (25.5)	200 (24.9)	169 (16.6)	52 (9.6)	0.249
Married, n (%)	275 (87.6)	668 (83.1)	823 (80.6)	415 (76.3)	0.159
**Clinical characteristics**					
SBP, mmHg, median (IQR)	132 (120-150)	130 (118-150)	130 (118-147)	130 (115-147)	0.089
DBP, mmHg, median (IQR)	80 (70-92)	80 (70-90)	80 (70-90)	80 (70-90)	0.051
Heart rate, b.p.m, median (IQR)	80 (68-96)	85 (72-100)	88 (74-102)	90 (76-105)	0.197
NYHA class, n (%)					0.536
II	109 (34.7)	152 (18.9)	85 (8.3)	28 (5.1)	
III	132 (42.0)	359 (44.7)	487 (47.7)	201 (37.0)	

© 2023 Huo X et al. *JAMA Network Open.*

IV	73 (23.3)	293 (36.4)	449 (44.0)	315 (57.9)	
**Medical history, n (%)**					
Hypertension	187 (59.6)	479 (59.6)	611 (59.9)	311 (57.4)	0.024
Atrial fibrillation	95 (30.3)	250 (31.4)	375 (37.2)	234 (43.2)	0.156
Myocardial infarction	83 (29.4)	188 (23.4)	228 (22.3)	137 (25.2)	0.055
Diabetes mellitus	93 (29.6)	226 (28.1)	338 (33.1)	197 (36.2)	0.100
COPD	46 (14.6)	127 (15.8)	207 (20.3)	115 (21.1)	0.104
Stroke	57 (18.2)	153 (19.0)	202 (19.8)	130 (24.0)	0.074
Anemia	35 (11.1)	102 (12.7)	191 (18.7)	119 (21.9)	0.174
New-onset HF	134 (42.7)	250 (31.1)	290 (28.4)	93 (17.1)	0.298
Current smoking	104 (33.1)	247 (30.7)	265 (26.0)	121 (22.2)	0.140
Depression	115 (36.6)	402 (50.0)	676 (66.2)	413 (75.9)	0.477
Cognitive impairment	64 (20.4)	214 (26.6)	316 (31.0)	197 (36.2)	0.194
**LVEF, %, median (IQR)**	44 (35-56)	43 (33-54)	43 (33-56)	43 (33-55)	0.098
**LVEF subtypes, n (%)**					0.106
HFrEF	124 (39.5)	384 (47.7)	482 (47.2)	264 (48.5)	
HFmrEF	74 (23.6)	163 (20.3)	191 (18.7)	105 (19.3)	
HFpEF	116 (36.9)	257 (32.0)	348 (38.8)	175 (32.2)	
**Biomarkers at admission, median (IQR)**					
NT-proBNP, ng/L	700 (220 -1632)	1125 (459-2371)	1427 (649-2990)	2008 (813-4369)	0.187

© 2023 Huo X et al. *JAMA Network Open.*

Sodium, mmol/L	140 (138-142)	140 (138-142)	140 (138-142)	139 (137-142)	0.113
Potassium, mmol/L	4.0 (3.8-4.3)	4.0 (3.7-4.3)	4.1 (3.7-4.5)	4.1 (3.7-4.4)	0.068
eGFR, mL/min/1.73m^2	76 (60-94)	77 (62-91)	72 (57-86)	70 (54-86)	0.167
**Medication at discharge, n (%)**					
ACEI/ARB	178 (56.7)	418 (52.0)	554 (54.3)	271 (49.8)	0.077
β-blockers	214 (68.2)	504 (62.7)	615 (60.2)	313 (57.5)	0.119
Aldosterone antagonists	190 (60.5)	497 (61.8)	673 (65.9)	371 (68.2)	0.095
**Quality of life at admission, median (IQR)**					
EQ-5D index	0.8 (0.8-1.0)	0.8 (0.7-0.8)	0.7 (0.5-0.8)	0.3 (0.1-0.6)	1.276
EQ-5D VAS	75 (70-80)	70 (60-80)	60 (50-70)	50 (40-65)	0.733

*SMDs less than 0.10 are considered small.

Abbreviation: HF: heart failure; IQR: interquartile range; SMD: standardized mean difference; SBP: systolic blood pressure; DBP: diastolic blood pressure; NYHA: New York Heart Association; COPD: chronic obstructive pulmonary disease; LVEF: left ventricular ejection fraction; HFrEF: heart failure with reduced ejection fraction; HFmrEF: heart failure with mildly reduced ejection fraction; HFpEF: heart failure with preserved ejection fraction; NT-proBNP: N-terminal pro-B type natriuretic peptide; eGFR: estimated glomerular filtration rate; ACEI: angiotensin-converting enzyme inhibitor; ARB: angiotensin receptor blocker; KCCQ-OS: Kansas city Cardiomyopathy Questionnaire overall summary score; EQ-5D: EuroQoL 5-dimension; VAS: visual analog scale;

© 2023 Huo X et al. *JAMA Network Open.*

eTable 5. Agreement Between NYHA Class and KCCQ-OS at Admission and 1 Month

Level of agreement	KCCQ-OS and NYHA class
**Admission, n (%)**	
-3 (KCCQ-OS 3 levels worse)	0
-2 (KCCQ-OS 2 levels worse)	28 (1.0)
-1 (KCCQ-OS 1 level worse)	286 (10.7)
0 (At the same level)	954 (35.6)
1 (KCCQ-OS 1 level better)	917 (34.2)
2 (KCCQ-OS 2 levels better)	425 (15.8)
3 (KCCQ-OS 1 levels better)	73 (2.7)
**1-month, n (%)**	
-3 (KCCQ-OS 3 levels worse)	1 (0)
-2 (KCCQ-OS 2 levels worse)	36 (1.3)
-1 (KCCQ-OS 1 level worse)	381 (14.2)
0 (At the same level)	1172 (43.7)
1 (KCCQ-OS 1 level better)	985 (36.7)
2 (KCCQ-OS 2 levels better)	102 (3.8)
3 (KCCQ-OS 1 levels better)	6 (0.2)

Abbreviation: NYHA: New York Heart Association; KCCQ-OS: Kansas City Cardiomyopathy Questionnaire overall summary score.

© 2023 Huo X et al. *JAMA Network Open.*

eTable 6. Factors Associated With Specific Directionality of Discordance Between NYHA Class and KCCQ-OS Categories

	KCCQ-OS better than NYHA class				NYHA class better than KCCQ-OS			
	Admission		1-month		Admission		1-month	
Variable	OR (95% CI)	*P* value	OR (95% CI)	*P* value	OR (95% CI)	*P* value	OR (95% CI)	*P* value
Female	0.74 (0.62-0.88)	<.001	0.79 (0.66-0.94)	0.01				
Employee	0.74 (0.60-0.93)	.01	0.77 (0.63-0.95)	0.01			1.95 (1.35-2.81)	<.001
Married			0.80 (0.64-0.99)	0.04				
Depression	0.52 (0.44-0.61)	<.001	0.47 (0.40-0.55)	<.001	1.72 (1.32-2.24)	<.001	1.68 (1.33-2.12)	<.001
Cognitive impairment							1.29 (1.03-1.62)	0.03
Hypertension	1.21 (1.03-1.43)	.02						
Smoking					0.55 (0.40-0.75)	<.001		
COPD	0.77 (0.63-0.94)	.01			1.80 (1.37-2.38)	<.001		
Stroke	0.79 (0.65-0.97)	.02						
Diabetes					1.35 (1.05-1.73)	.02		
Anemia			0.67 (0.53-0.84)	<0.001				
New-onset HF	1.23 (1.03-1.47)	.02					0.75 (0.58-0.97)	.03
LVEF subtypes*	0.77 (0.64-0.83)	.002			1.47 (1.12-1.94)	.05	1.58 (1.24-2.02)	<.001

Note: Odds ratio>1 suggests a greater likelihood of discordance between KCCQ-OS and NYHA class, and odds ratio<1 suggests the reverse is true (e.g., for female 0.74 in the upper left cell, this means that females are less likely than males to have a KCCQ better than the NYHA class

© 2023 Huo X et al. *JAMA Network Open.*

at admission)

* LVEF subtypes indicate HFpEF vs. HFrEF

Abbreviation: OR: odds ratio; CI: confidence interval; NYHA: New York Heart Association; HF; heart failure; COPD: chronic obstructive pulmonary disease; KCCQ-OS: Kansas City Cardiomyopathy Questionnaire Overall score; LVEF: left ventricular ejection fraction; HFrEF: heart failure with reduced ejection fraction; HFpEF: heart failure with preserved ejection fraction.

© 2023 Huo X et al. *JAMA Network Open.*

eTable 7. Correlations Among NYHA Class and KCCQ Measurements

	KCCQ-OS	KCCQ-PLS	KCCQ-SFS	KCCQ-QOLS	KCCQ-SLS
**Admission**					
NYHA class	r_s=0.26 p=<.001	r_s=0.18 p=<.001	r_s=0.23 p=<.001	r_s=0.25 p=<.001	r_s=0.21 p=<.001
**1-month**					
NYHA class	r_s =0.54 p=<.001	r_s=0.50 p=<.001	r_s=0.46 p=<.001	r_s=0.38 p=<.001	r_s=0.48 p=<.001

Abbreviation: NYHA: New York Heart Association; KCCQ-OS: Kansas City Cardiomyopathy Questionnaire overall summary score; KCCQ-CSS: Kansas City Cardiomyopathy Questionnaire clinical summary score; KCCQ-PLS: Kansas City Cardiomyopathy Questionnaire physical limitation score; KCCQ-SFS: Kansas City Cardiomyopathy Questionnaire symptom frequency score; KCCQ-SLS: Kansas City Cardiomyopathy Questionnaire social limitation score; KCCQ-QOLS: Kansas City Cardiomyopathy Questionnaire quality of life score.

© 2023 Huo X et al. *JAMA Network Open.*

eTable 8. Association Between Changes in Each Domain of KCCQ-OS and Clinical Outcomes

	KCCQ-SFS: 5-point improvement		KCCQ-PLS: 5-point improvement		KCCQ-SLS: 5-point improvement		KCCQ-QOLS: 5-point improvement	
	Improvement vs no improvement HR (95% CI)	*P* value	Improvement vs no improvement HR (95% CI)	*P* value	Improvement vs no improvement HR (95% CI)	*P* value	Improvement vs no improvement HR (95% CI)	*P* value
**All-cause death**								
**1-year**								
unadjusted	0.59 (0.46-0.76)	<.001	0.57 (0.45-0.72)	<.001	0.58 (0.46-0.73)	<.001	0.69 (0.55-0.88)	0.003
adjusted	0.59 (0.46-0.77)	<.001	0.56 (0.44-0.71)	<.001	0.63 (0.50-0.80)	<.001	0.71 (0.56-0.91)	0.01
**4-year**								
unadjusted	0.86 (0.75-0.99)	.04	0.79 (0.70-0.90)	<.001	0.71 (0.63-0.80)	<.001	0.93 (0.83-1.06)	.28

© 2023 Huo X et al. *JAMA Network Open.*

adjusted	0.87 (0.75-1.00)	.05	0.82 (0.73-0.93)	.002	0.77 (0.68-0.87)	<.001	0.99 (0.87-1.12)	.84
**CV death or HF hospitalization**								
**1-year**								
unadjusted	0.73 (0.63-0.84)	<.001	0.74 (0.66-0.85)	<.001	0.66 (0.58-0.75)	<.001	0.69 (0.61-0.79)	<.001
adjusted	0.71 (0.61-0.82)	<.001	0.74 (0.65-0.84)	<.001	0.71 (0.62-0.80)	<.001	0.70 (0.61-0.79)	<.001

Unadjusted and adjusted hazard ratios indicate the risk of clinical outcomes associated with 5-point improvement in each domain of Kansas City Cardiomyopathy Questionnaire. Models were adjusted for age, sex, educational attainment, employment, smoking, depression status, cognitive function, systolic blood pressure, N-terminal pro-B type natriuretic peptide, estimated glomerular filtration rate, serum sodium, serum potassium, left ventricular ejection fraction subtypes, atrial fibrillation, diabetes, chronic obstructive pulmonary disease, myocardial infarction, stroke, anemia, prior heart failure, cardiac resynchronization therapy, implantable cardioverter-defibrillator, and post-discharge medications.

Abbreviation: HR: hazard ratio; CI: confidence interval; CV death: Cardiovascular death; HF: heart failure; KCCQ-OS: Kansas City Cardiomyopathy Questionnaire overall summary score; KCCQ-SFS: Kansas City Cardiomyopathy Questionnaire symptom frequency score.; KCCQ-PLS: Kansas City Cardiomyopathy Questionnaire physical limitation score; KCCQ-SLS: Kansas City Cardiomyopathy Questionnaire social limitation score; KCCQ-QOLS: Kansas City Cardiomyopathy Questionnaire quality of life score.

© 2023 Huo X et al. *JAMA Network Open.*

eFigure 2. Sensitivity Analysis of the Association Between Change in KCCQ-OS and Clinical Outcomes

Clinical outcomes	Imorovement vs no improvement Hazard Ratio (95% CI)	P value
**KCCQ-OS: 10-point improvement**		
**All-cause death**		
**1-year**		
Unadjusted	0.57 (0.45-0.73)	<0.001
Adjusted	0.60 (0.47-0.76)	<0.001
**4-year**		
Unadjusted	0.78 (0.69-0.88)	<0.001
Adjusted	0.83 (0.74-0.95)	0.004
**CV death or HF rehospitalization**		
**1-year**		
Unadjusted	0.64 (0.57-0.73)	<0.001
Adjusted	0.66 (0.58-0.75)	<0.001
**KCCQ-OS: 20-point improvement**		
**All-cause death**		
**1-year**		
Unadjusted	0.58 (0.45-0.74)	<0.001
Adjusted	0.58 (0.45-0.75)	<0.001
**4-year**		
Unadjusted	0.77 (0.68-0.87)	<0.001
Adjusted	0.80 (0.70-0.90)	<0.001
**CV death or HF rehospitalization**		
**1-year**		
Unadjusted	0.68 (0.60-0.78)	<0.001
Adjusted	0.69 (0.61-0.78)	<0.001

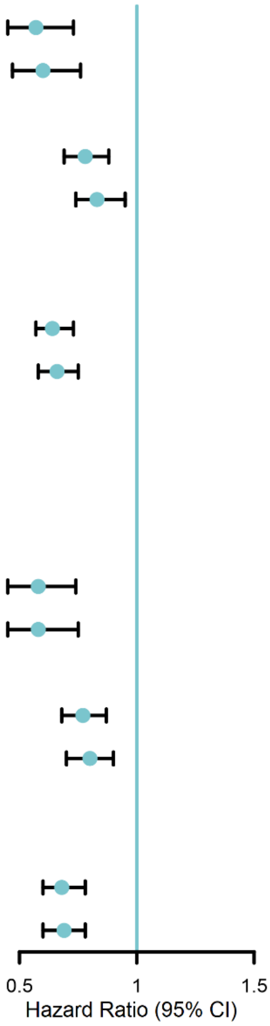

Unadjusted and adjusted hazard ratios indicate the risk of clinical outcomes associated with 10-point and 20-point improvement in Kansas City Cardiomyopathy Questionnaire overall summary score. Models were adjusted for age, sex, educational attainment, employment, smoking, depression status, cognitive function, systolic blood pressure, N-terminal pro-B type natriuretic peptide, estimated glomerular filtration rate, serum sodium, serum potassium, left ventricular ejection fraction subtypes, atrial fibrillation, diabetes, chronic obstructive pulmonary disease, myocardial infarction, stroke, anemia, prior heart failure, cardiac resynchronization therapy, implantable cardioverter-defibrillator, and post-discharge medications.

© 2023 Huo X et al. *JAMA Network Open.*

Abbreviation: KCCQ-OS: Kansas City Cardiomyopathy Questionnaire overall summary score; CV death: cardiovascular death; HF: heart failure; CI: confidence interval.

© 2023 Huo X et al. *JAMA Network Open.*

eFigure 3. Subgroup Analysis of the Association Between Change in NYHA Class and KCCQ-OS With 4-Year All-Cause Mortality

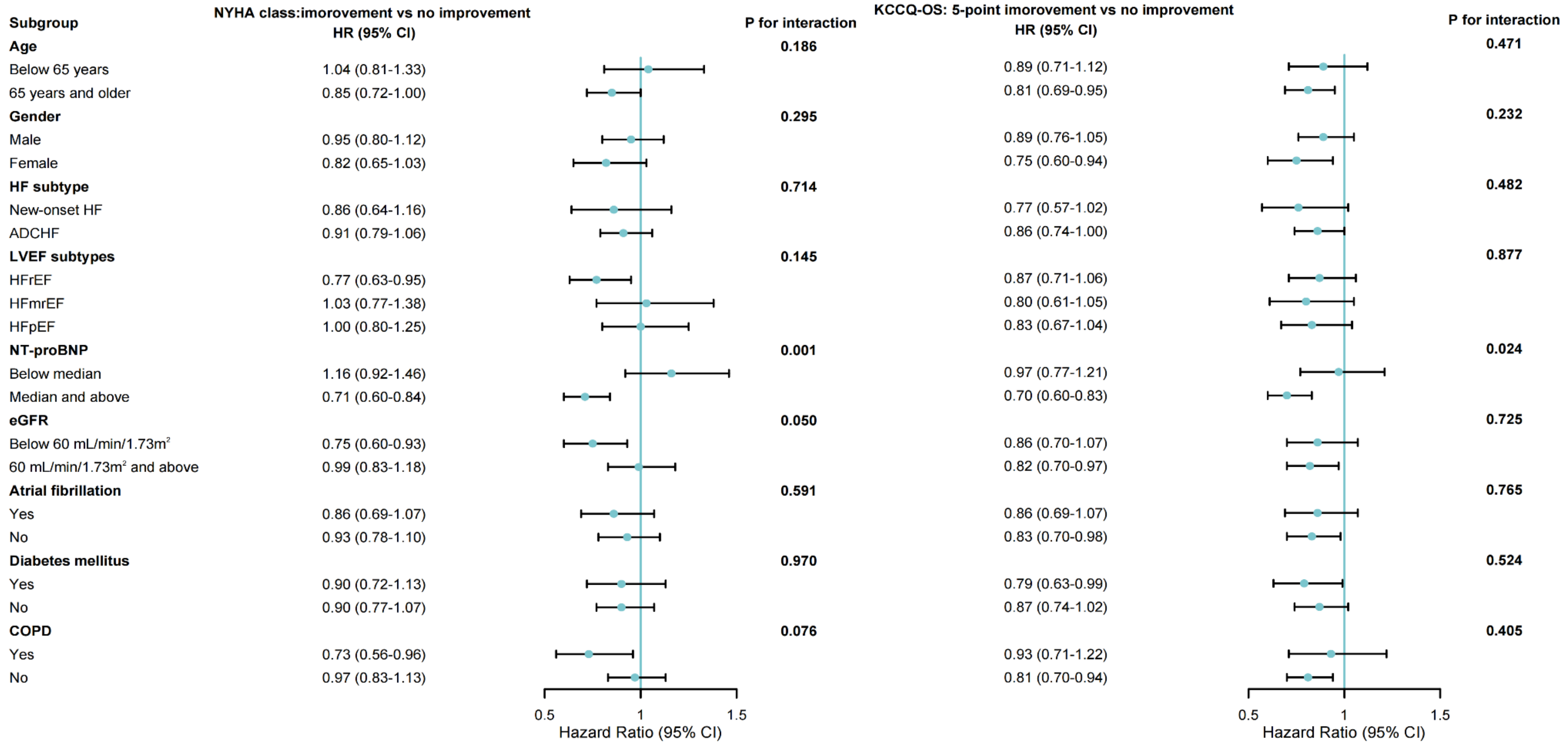

Hazard ratios indicate the risk of clinical outcomes associated with improvement in New York Heart Association class and Kansas City Cardiomyopathy Questionnaire overall summary score in different subgroups. Models were adjusted for age, sex, educational attainment,

© 2023 Huo X et al. *JAMA Network Open.*

employment, smoking, depression status, cognitive function, systolic blood pressure, N-terminal pro-B type natriuretic peptide, estimated glomerular filtration rate, serum sodium, serum potassium, left ventricular ejection fraction subtypes, atrial fibrillation, diabetes, chronic obstructive pulmonary disease, myocardial infarction, stroke, anemia, prior heart failure, cardiac resynchronization therapy, implantable cardioverter-defibrillator, and post-discharge medications.

Abbreviation: NYHA: New York Heart Association; KCCQ-OS: Kansas City Cardiomyopathy Questionnaire overall summary score; HF: heart failure; ADCHF: acutely decompensated chronic heart failure; NT-proBNP: N-terminal pro-B type natriuretic peptide; eGFR: estimated glomerular filtration rate; HF: heart failure; LVEF: left ventricular ejection fraction; HFrEF: heart failure with reduced ejection fraction; HFmrEF: heart failure with mildly reduced ejection fraction; HFpEF: heart failure with preserved ejection fraction; COPD: chronic obstructive pulmonary disease; HR: hazard ratio; CI: confidence interval.

© 2023 Huo X et al. *JAMA Network Open.*
