# Supplementary material for: New York Heart Association Class and Kansas City Cardiomyopathy Questionnaire in Acute Heart Failure
Source: JAMA Netw Open. 2023 Oct 24;6(10):e2339458. doi: 10.1001/jamanetworkopen.2023.39458 (PMC10599126; doi:10.1001/jamanetworkopen.2023.39458)
Supplement: Supplement 2. — Data Sharing Statement [file jamanetwopen-e2339458-s002.pdf]

Data Sharing Statement

Huo. New York Heart Association Class and Kansas City Cardiomyopathy Questionnaire in Acute Heart Failure. *JAMA Netw Open*. Published October 24, 2023.
doi:10.1001/jamanetworkopen.2023.39458

Data
Data available: No
